# Effectiveness of conventional treatment using bulk-fill composite resin versus Atraumatic Restorative Treatments in primary and permanent dentition: a pragmatic randomized clinical trial

**DOI:** 10.1186/s12903-016-0260-6

**Published:** 2016-08-02

**Authors:** Isabel Cristina Olegário, Daniela Hesse, Marcelo Bönecker, José Carlos Pettorossi Imparato, Mariana Minatel Braga, Fausto Medeiros Mendes, Daniela Prócida Raggio

**Affiliations:** 1 Department of Paediatric Dentistry, School of Dentistry, Faculdade de Odontologia da Universidade de São Paulo, Av. Lineu Prestes, 2227, São Paulo, SP 05508-000 Brazil; 2 Department of Cariology, Endodontics and Pedodontology, Academic Centre for Dentistry Amsterdam (ACTA), Amsterdam, The Netherlands

**Keywords:** Atraumatic Restorative Treatment, Composite resin, Glass ionomer cement, Randomized clinical trial, Cost effectiveness analysis

## Abstract

**Background:**

Clinical studies are being conducted in less strict conditions in order to establish an adequate scientific basis for decision making. The aim of this pragmatic randomized clinical trial is to evaluate the effectiveness of single and multiple-surfaces restorations performed following the Atraumatic Restorative Treatment (ART) premises compared with Conventional Treatment (CT) using bulk fill composite restorations in primary and permanent teeth.

**Methods/design:**

A total of 1,214 5-to-13 year-old children with at least one single or multiple-surface dentin caries lesion in primary or permanent molars will be selected in public schools of Barueri-SP, Brazil. The participants will be randomly assigned into 2 groups: CT (caries removal with bur and restoration performed with Scotchbond™ Universal Adhesive system associated with Filtek Bulk Fill – 3 M/ESPE) and ART (Caries removal with hand instruments and restoration with high viscosity glass ionomer cement Ketac Molar Easy Mix – 3 M/ESPE). Ten untrained dentists will perform the treatment in in dental offices located at public schools. The restorations will be evaluated after 6, 12 and 24 months by an independent trained and calibrated examiner. The restoration and tooth survival, the cost-effectiveness analysis between the two groups and the operators’ preferences regarding the techniques will be also evaluated. Kaplan-Meier survival analysis and log-rank test will be applied for the restoration and tooth survival. All the average event rates in the two groups will be modelled and compared with a Cox proportional hazard shared frailty model since there is an operator-cluster effect. The significance level for all analyses will be 5 %.

**Discussion:**

Our hypothesis is that despite similar expected effectiveness between ART using high viscosity GIC and conventional treatment using bulk fill composite resin when treating single or multiple-surface in posterior primary and permanent teeth, ART will present superior cost-effectiveness. The results of this trial will support decision-making by clinicians and policy makers.

**Trial registration:**

NCT02568917. Registered on May 10th 2015.

**Electronic supplementary material:**

The online version of this article (doi:10.1186/s12903-016-0260-6) contains supplementary material, which is available to authorized users.

## Background

Currently, both in medicine and dentistry, clinical studies are being conducted in less strict conditions, closer to those found in practice in order to establish an adequate scientific basis for decision making, the so-called pragmatic clinical trials. Trough those studies design, a closer representation of the characteristics of the patient and how the intervention will take place in real clinical practice is expected [1]. However, few pragmatic studies were conducted in dentistry, especially when the primary outcome is the effectiveness of restorative treatment [2, 3].

The Atraumatic Restorative Treatment (ART) has been considered an innovative, painless and minimally invasive treatment for the management of caries [4, 5]. Initially, this treatment was recommended to populations in which the technical and operational conditions were unfavourable [6]. However, ART has been proven to be a high quality and reliable approach in the management of dental caries, and became, therefore, suitable for all patients, regardless of the economic and social situation [7, 8]. Allied to these indications, the fact anesthesia, rubber dam and rotary instruments are not required, stimulated the ART use in the public and private health systems compared to conventional treatment (CT).

In order to reduce the clinical time of the conventional restorative procedure, a new concept in composite resins is emerging [9]. The “bulk fill” composite which intended to reduce the polymerization shrinkage stress (main drawback of composite resin), has been gaining strength in the dental market. Thanks to its technical ease of use, since the restorations can be performed by a single increment of resin (up to 4 mm), this material can be an alternative to restorations in public health, in which rarely rubber dam is employed. As the material is inserted in one increment, the time expended to perform the treatment can be shortened, thus reducing the chance of contamination and possibly increasing restoration longevity.

When it comes to the real applicability of ART compared to CT using composite resin, there is little evidence regarding both the longevity of these restorations and the preference of dentists on the use of each technique [2, 3].

Due to the need of establishing the best scientific evidence about restorative treatment, the aim of this pragmatic clinical trial is to evaluate the effectiveness of single and multiple-surface ART-restorations performed compared to the CT in primary and permanent molars.

## Methods/Design

The present protocol follows the guidelines of the Standard Protocol Items: Recommendations for Interventional Trials (SPIRIT) (Additional file 1).

### Ethical aspects and registration

This clinical trial was approved by the Local Ethics Committee of Research in Humans (#1.293.897) and registered in the database for registration of clinical studies Clinicaltrials.gov (registration no. NCT02568917).

The study will be conducted in Barueri, a city in the state of São Paulo, Brazil. Informed consent will be obtained from children’s parents or guardians before participation in the study and each child must also assent to participate.

All participants will be encoded by a number in order to guarantee information confidentiality. In case of files containing identifiable data, those will be stored in locked filing cabinets and final dataset will be available for inspection under coordinator’s endorsement. Thus, there is no Data Monitoring Committee. Only researchers will have access to participants’ information. Independent surveillance of trial data collection, management and analysis is undertaken by the main investigator (ICO) who has overall responsibility for the study and is in charge of the data. The collected data will be subject to audition by the coordinator; by raising data queries if necessary. This protocol has been peer-reviewed by the funding agency.

The adverse events related to the CT or ART (groups investigated in this trial) are those related to dental treatment. These effects are similar to those found during a conventional treatment performed in the pediatric dentistry clinical practice. The operators of this project will conduct all dental treatment needs of the children participating in this trial.

### Study design

The design of this project comprises different dentitions (primary and permanent teeth) and different surfaces (single or multiple-surface).

**Fig. 1:**
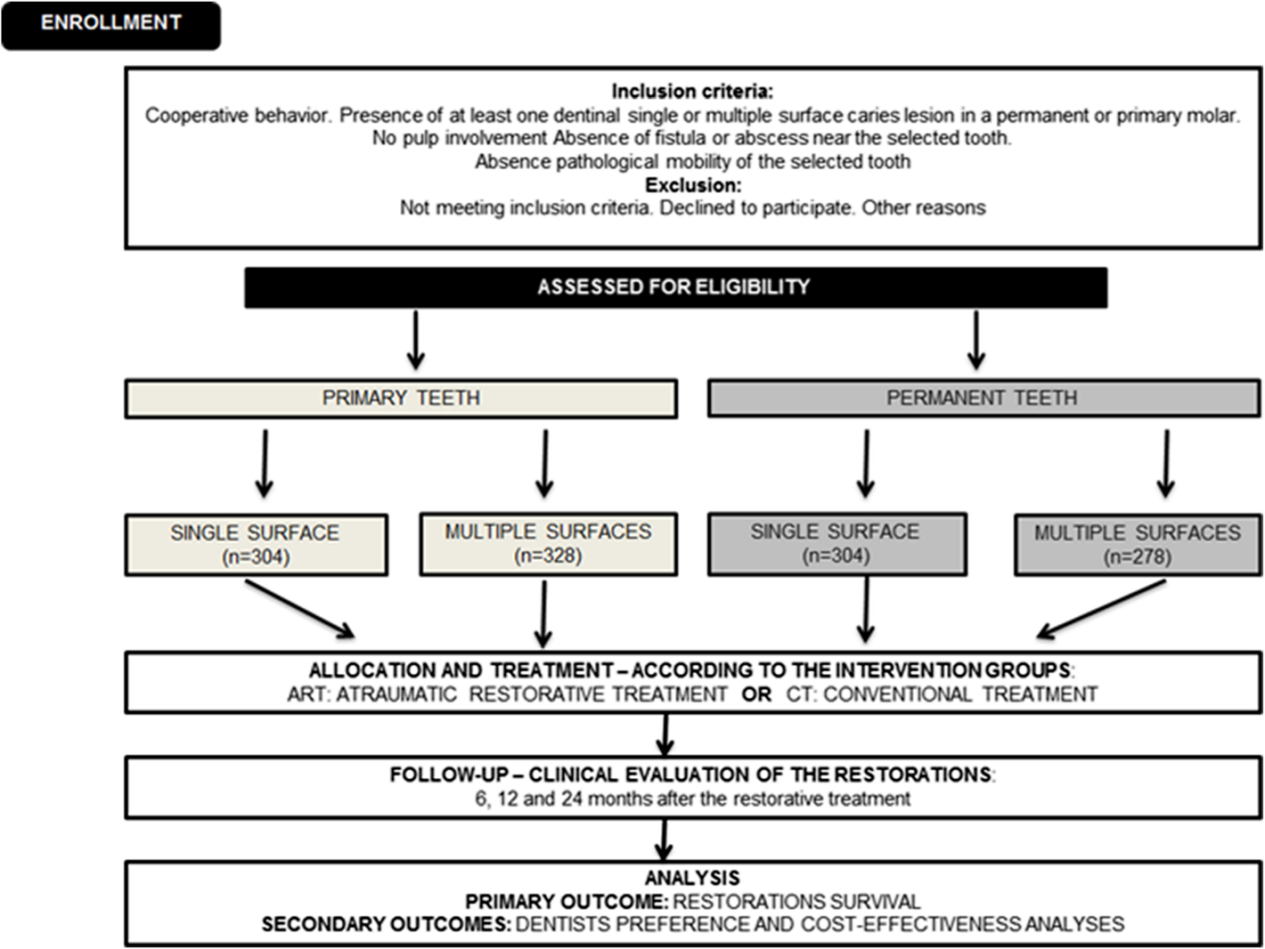
Clinical trials flowchart

This is a pragmatic, two-arm, parallel group, patient-randomized trial which has 10 dentists as operators in dental offices located at public schools. The schedule of enrolment, interventions, and assessments diagram is showed in Fig. 1.

### Sample description

Since single and multiple-surfaces cavities are going to be restored, both in primary and permanent teeth, and the difference expected between those groups are not the same, four sample size calculations were done.

The sample size calculation was performed using the website statstodo.com based on the primary outcome - treatment survival, using log-rank test in survival analysis. For the single surface, the calculation was based in an absolute difference between the survival rates of CT vs. ART of 15 % while for the multiple-surfaces, the absolute difference considered was 20 %. This involved a two-tailed test based on survival rate reported for ART [10] after 2 years of follow- up, α of 5 and power (strength) of 80 %. The sample size was increased 20 to compensate for possible losses during the study and 40 % to compensate operators-cluster effect. This gave a final sample size of 1214 teeth (described in Table 1).

**Table 1:** Sample size calculation description

Tooth type	Surface	Survival Rate reported for ART (2 years)	Sample size	+20 % possible losses	+40 % operator-cluster effect
Permanent	Single surface	93	181	253	304
	Multiple surfaces	62	165	231	278
Primary	Single surface	93	181	253	304
	Multiple surfaces	41	195	273	328
				Total	1, 214

### PRECIS (Pragmatic-Explanatory Continuum Indicator Summary) domains

#### Elegibility/Recruitment/Setting

The participants of the trial are those who would receive dental treatment in a usual care. No extra effort will be made to recruit more participants. All children that have at least one single or multiple-surface caries lesion in a primary or permanent posterior tooth and who seek for treatment in one of the participating centers are eligible for this trial.

Only the children whose parents or legal guardians signed the informed consent and who assent to be part of the study will be included in the research. All phases of this trial will be carried out in the dental clinic located at public schools. As radiographic facilities are not available in this setting, only clinical diagnosis will be used.

As this trial aims to evaluate the restoration and tooth survival, the tooth of interest should not present any associated fistula, abscess, pulp exposure, historical of spontaneous dental pain or mobility.

Even when children do not present a cooperative behavior, they will be included in this trial, as it is a common problem in pediatric dentistry clinical practice. Other variables like size of the cavity, approximal or occlusal contact and caries experience will also not be considered as exclusion criteria. All data will be recorded and further analysed to manage if there is any influence of those clinical factors on the survival rate of the restorations.

Recruitment will take place from March to November 2016. Each participant will be enrolled in the study for about 25 months: 1 month for RCT diagnosis and treatment, followed by a 24-month observational period. Details are illustrated in Fig. 1.

### Organization intervention/Operators

The operators of this study will be 10 dentists who work in the dental offices located at the public schools of Barueri. All operators will receive only a guideline of how to prepare, restore and finalize the restoration according to the original procedures of ART [2]. Regarding the procedures in the CT group, all the dentists will receive explanations about the preparation of the cavity according to partial caries removal philosophy and also about the manufactures’ specifications of the materials used. Since it is a pragmatic clinical trial, operators with different clinical expertise will be selected and no training will be performed for both groups.

### Intervention flexibility – delivery/Adherence

The intervention will be delivered and the flexibility of the trial will be similar to the usual care. The treatments will be done by the dentists of the municipality during their normal working setting.

The patient will be invited to participate of the research since they have matched the inclusion criteria. No extra encouragement to adhere to the study will be done.

### Participant selection

The criteria for caries presence will be the World Health Organization [11] and the DMFT and dmft will be recorded [12]. The biofilm evaluation according to the criteria proposed by Löe (1972) [13] and the gingival health [14] will be held before the restoration (baseline).

### Randomization

The randomization lists were computer generated (www.randomization.com), based on randomly permuted blocks of varying size (2, 4 or 6, randomly sampled with equal probability) and stratified for operator (1–10), dentition (primary/permanent) and surface (single/multiple). Opaque, sealed and sequentially numbered envelopes will be used to randomize the participants into the treatment groups (ART and CT).

### Study groups

Participants will be randomly assigned into two different groups:

- Atraumatic Restorative Treatment (ART) (control): ART-restorations using high viscosity glass ionomer cement (GIC) Ketac Molar EasyMix (3 M/ESPE) with manual dosage and hand-mix powder-liquid.
- Conventional Treatment (CT) (experimental): composite resin restoration, Scotchbond™ Universal Adhesive (3 M/ESPE) and the Filtek Bulk Fill composite resin (3 M/ESPE).

### Interventions

- Atraumatic Restorative Treatment (ART) using Glass Ionomer Cement (GIC)
  The treatments from ART group will be performed according to the ART guidelines as described by Frencken et al. [15]. The isolation of the operating site will be done with cotton wool. The cavity will be opened with a dental hatchet and enamel cutters. Regarding the dentin caries removal, only will be removed the soft and completely demineralized dentine with hand excavators.
  For the dentin conditioning, will be used the first drop of 11.5 % polyacrilic acid with a microbrush during 15 s. Then, the cavity will be washed with three cottons moistened with water and dry using other three cotton pellets.
  After this step, the dosage (1:1 powder-liquid ratio) and hand mixing of the material will be made. The material only can be applied while it remains glossy. The GIC application will be done with a#1 spatula followed by finger pressure using petroleum jelly. In occlusal-proximal cavities, an adapted matrix with a wooden wedge must be used to provide an appropriate contour to the restoration.
  After the initial setting, the occlusion will be checked with articulating paper. A new layer of petroleum jelly should be applied on the surface of the restoration after removing the excess. The dentists will give instruction to the patient to not eat solid food for one hour.
- Conventional Treatment (CT) using Bulk Fill composite resin
  The teeth in this group will be treated in a conventional way, which dentists are used to carry out followed by restoration with composite resin. As a routine program in public health center of Barueri, no rubber dam will be used.
  Instructions about the removal of carious tissue will be given, using a spherical carbide drill at low speed under refrigeration or using hand instruments, according to the operator preference.
  After the preparation of the cavity, no cavity conditioner will be applied, since the adhesive system used (Scotchbond™ Universal Adhesive - 3 M ESPE) allows the self-etch technique.
  The restoration will be performed using composite resin (Filtek Bulk Fill – 3 M ESPE). If the cavity size is bigger than 4 mm, two increments must be applied.
  For light curing, both for the adhesive system and the composite resin, the same light equipment will be used. If necessary, finishing bur will be used for adjustments.

### Follow-up

All the evaluations will be carried out by one single trained and calibrated evaluator (Kappa >0.70). The children that participated in the study will be localized by the national education system, regarding the school that they study, classroom and phone number.

An independent and calibrated evaluator will go to the school that the child studies after 6, 12 and 24 months to perform the evaluations.

The evaluation of the restorations will be held according to Roeleveld et al. [16] (Additional file 2). The width and depth of marginal defects, the surface wear and the excess or lack of material will be measured using the CPI periodontal probe which has a ball-shaped end-point of 0.5 mm in diameter.

### Outcomes

As a pragmatic trial, the outcomes must be relevant to the patient. So, the primary outcome of this study is the longevity of single and multiple-surfaces ART-restorations compared to Conventional Treatment (CT) in primary and permanent molars.

As secondary outcomes we will evaluate the technical preference assessment by dentists using an adapted and translated questionnaire [3] (Additional file 3) and the evaluation of the cost-effectiveness of the two techniques applied. The longevity of the tooth, in case of primary teeth will also be evaluated.

(I) Longevity of the restoration
  Treatment longevity will be evaluated after 6, 12, 18 and 24 months by one trained and calibrated examiner. The intra-examiner and inter-examiner agreement will be calculated using the Cohen’s Kappa test and only scores above 0.7 will be accepted.
  Following on the Roeleveld et al. [16] criteria, it will be considered “success” only the restorations that receive the scores 00 and 10. The scores 11–40 will be considered “failure”. The scores 50, 60, 70 and 90 will not be assessed in the analysis of the success of the restoration.
(II) Longevity of the tooth
  This outcome will be calculated only for the primary teeth included in this research. Intact restorations and the ones with a minor failure of the restoration (scores 00 to 30) will be considered as “success”, using the same criteria mentioned above [16]. Only the restored teeth that presents symptoms of pulp inflammation or need extraction (scores 40 and 50) will be considered as “failure”, since it cannot be considered as a successful treatment for the tooth.
  The main objective of a restoration is to provide a better condition for the patient by improving oral hygiene, inactivating the caries process, and returning the masticatory function of that tooth, so the scores considered “minor failures” will not be considered failure for the tooth [17].
(III) Cost-effectiveness
  To calculate the direct and indirect costs of the procedures, it will be taken into account the time spent on each treatment, including the treatment and the follow-up visits. The reference group to do the cost analysis will be the ART. The time spent in each session will be timed by a dental assistant (which is not participating in diagnostic tests or treatment) in order to input the cost-effectiveness analysis of the methods used in the research. All the information will be registered in predetermined sheets the specifications and quantity of all materials used.
  For the calculation of direct costs, it will also be considered the prices of materials used in each procedure based on an actual dental market value converted in US Dollars and obtained by the mean values from different dental stores for the referred products. In order to calculate the Professional Cost, we will calculate the time spent in each session and convert it in hours and multiply it by the medium income of the dentist per hour as related by the Brazilian Ministry of Labor and Employment ($36.23).
  To estimate the procedure cost, it will be considered both variable cost, which includes electricity and equipment depreciation, and material costs [18, 19]. To calculate the equipment depreciation (peripherals, dental chair and instrumental), we will consider their costs, the life span of five years and a monthly use of 160 h, using an estimate value per hour of $1.81.
(IV) Preference of the treatments by dentists
  The preference of dentists will be evaluated at the end of the operative phase. Thus, we aim to identify which is the preferred procedure by professionals. To evaluate this outcome, a questionnaire composed of six items will be applied.
  This questionnaire was adapted from the study of Pani et al. [3], which evaluated the preference of students with respect to composite and silver amalgam. The questionnaire was translated from English to Portuguese by a Brazilian dentist who is fluent in both languages and was adapted for comparing the composite resin and the glass ionomer cement used according to the ART. This questionnaire will be administered before and after the research for the dentists (Additional file 3).

### Data analysis

Data will be analyzed according to the protocol principle. First, to compare treatments’ and teeth longevity, both Kaplan-Meier survival analysis and Log-rank test will be applied. The association between restoration longevity and caries experience or type of cavity will be evaluated using Cox Regression.

As we have a cluster-effect related to the numbers of operators of this trial and its contextual variables (such as age, work experience and technique preference), the average event rates in the two groups over the two year study period will be then modelled and compared with a “shared frailty” model. This model is an extension of the Cox proportional hazard model that includes a frailty term (a statistical expression for random effect, unrelated to the clinical concept of frailty) to take dependency of events within a contextual (correlation between different falls by the same operator) into account. All participants will be included in the model until their last time point, which means that the model will use all available data right up to the time of withdrawal or trial completion. For each outcome, we will present the hazard ratios and 95 % confidence intervals for the intervention, adjusted by operator.

The variance analysis will be used to compare groups in relation to the cost after determining data normality through the Kolmogorov-Smirnov test. Poisson analysis will be used to assess the association between the treatment group, the preference of professionals and the cost of treatments. The significance level will be set in 5 %.

## Discussion

The aim of this study is to determine the effectiveness of two different treatments most used in clinical practice: ART using high viscosity GIC and CT using bulk fill composite resin and a universal adhesive.

**Fig. 2:**
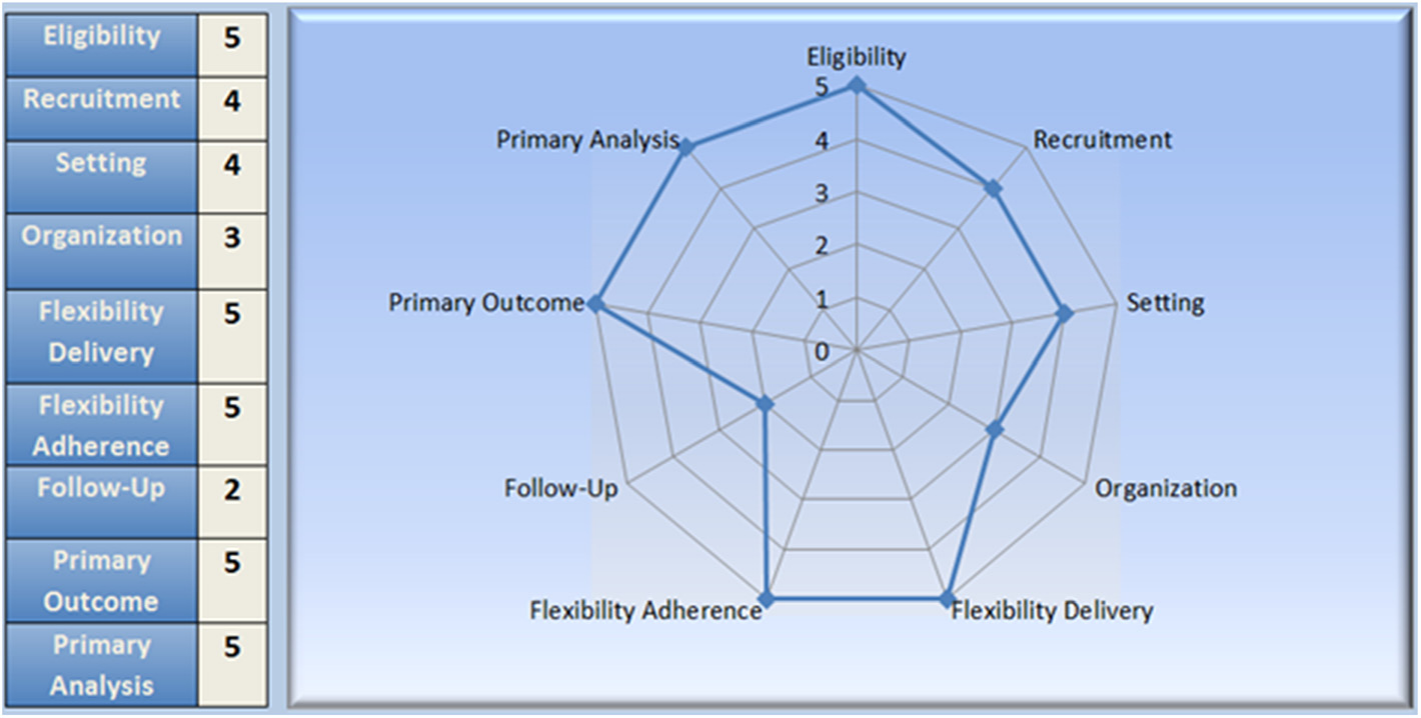
PRECIS domains diagram

No study is completely pragmatic, nor is it completely explanatory. However in order to overcome the drawbacks often associate with this kind of study design, the PRECIS (Pragmatic-Explanatory Continuum Indicators) tool was created. The Fig. 2 shows the multiple dimensions and how pragmatic the project is regarding all domains: eligibility, recruitment, setting, organization, flexibility delivery and adherence, follow-up, primary outcome and primary analysis.

As a pragmatic study, the conditions reported in this protocol are similar to those in real dental practice and the operators are dentists who work for the public health system. The decision to not perform any training for the operators is to maintain a closer representation of the dentists’ reality in dental practice, since they have different backgrounds of undergraduate learning. In that way, the operators only will receive one guideline on how to prepare, restore and finalize the restoration according to the original procedures of ART [2]. Regarding the procedures performed in the CT group, as all the dentists are used to do composite resin in their daily clinical practice, they will receive only some information about the manufacturer specifications for using these new materials. Due the evident differences between the techniques and materials used in each group presented in this trial, it is not possible to blind the patients, operators or evaluators.

Few clinical studies are reported in the literature using a bulk fill composite resin [9, 20] and to the best of our knowledge this is the first study to use this material also for restoring primary teeth. As this material can be used in a single increment until 4 mm allied to the use of a universal adhesive, which allows the self-etch technique, the clinical time may be shortened, reducing the contamination probability. As no rubber dam is available in public health dental practice in Brazil, the use of these materials could be an answer to improve the conventional treatment longevity.

In cases where the child has more than one eligible cavity in the same dentition and surface (for example: two single-surface cavities in a primary molar in the same child), only one cavity will be randomly selected. In case of different teeth (permanent and primary) or different surfaces (single or multiple-surface), more than one tooth can be selected for the research, since the analysis of each group will be done separately (for example: one single-surface in a primary molar and one single surface in a permanent molar in the same child). In this case, each tooth will be randomly allocated to the intervention group since the analyses are going to be done separately according to dentition and surface.

In order to evaluate the preferences of dentists and measure a professional-centered outcome, a questionnaire composed of sixteen items will be applied [3]. It aims to evaluate factors influencing dentists’ preference of amalgam or composite for posterior dental restorations, which can influence the quality of the restorations.

The growing use of cost-effectiveness analysis (CEA) to evaluate the costs and health effects of specific interventions is dominated by studies of prospective new interventions compared to current practice [21]. The dental treatments (ART and CT) are daily performed during the normal clinical practice, but maybe with different materials (there is a large range of materials available in Brazilian market). In order to standardize the materials that will be used in this trial, we received a donation from the manufacturer. There is no conflict of interests, and the results will be published in good quality journals.

The patients of this trial will be children who study in the school where the dental office is located. Even it is not the best pragmatic way to perform the follow-up, the evaluation will be done by a single evaluator, in a school setting, in order to reduce drop-outs during the follow-up. This evaluation system is possible since we can locate the child aided by a national student’s data website [22].

Regarding our main outcome, to evaluate the survival rate of the ART and CT restorations, we chose the Roeleveld criteria [16], that allows splitting the evaluations in minor and major failure [23], therefore the survival of the tooth (in case of primary dentition) can also be calculated. The objective of a restoration is to provide the patient a condition for arresting caries lesion and avoid pulp problems or extractions. For that reason, studies should focus on the survival of the restored teeth and not remain limited to the minor failures of restoration survival [24, 25].

The ART is already well described in the literature, both in primary and permanent teeth [10, 26, 27] and well accepted by dentist due to its ease of use. In another hand, little is known about bulkfill composite resin, which can also be used as an aesthetic restoration and in one single increment. In that way, this pragmatic study will contribute to improve the knowledge about the ART and CT for restoring single or multiple surface carious lesions in primary and permanent dentition.

## Trial status

This trial is recruiting patients.

## Abbreviations

ART, Atraumatic Restorative Treatment; CONSORT, consolidated standards of reporting trials; CT, conventional treatment; DMFT, decayed, missing, filled teeth in permanent teeth; dmft, decayed, missing, filled teeth in primary teeth; GIC, glass ionomer cement; mm, millimeter; PRECIS, Pragmatic-Explanatory Continuum Indicator Summary; WHO, World Health Organization

## Additional files

Additional file 1: SPIRIT Checklist. (DOC 120 kb) Additional file 2: Evaluation Criteria modified from Roeleveld et al. 2006. (DOCX 15 kb) Additional file 3: Questionnaire adapted from Pani et al. (2014) [3]. (DOCX 18 kb)

## References

[1] Patsopoulos NA (2011). A pragmatic view on pragmatic trials. Dialogues Clin Neurosci.

[2] Frencken JE, van’t Hof MA, Taifour D, Al-Zaher I (2007). Effectiveness of ART and traditional amalgam approach in restoring single-surface cavities in posterior teeth of permanent dentitions in school children after 6.3 years. Community Dent Oral Epidemiol.

[3] Pani SC, Abbassi MFA, Saffan AD, Sumait MAA, Shakir AN (2014). Factors influencing Saudi dental students’ preference of amalgam or composite for posterior dental restorations. S J Oral Sci.

[4] Ericson D, Kidd EAM, McComb D, Mjor I, Noack MJ (2003). Minimally invasive dentistry—concept and techniques in cariology. Oral Health Prev Dent.

[5] Czarnecka B (2006). The use of ART technique in modern dental practice: a personal view. J Dent.

[6] Smales RJ, Yip HK (2002). The atraumatic restorative treatment (ART) approach for the management of dental caries. Quintessence Int.

[7] Ismail A (1996). Minimal intervention techniques for dental caries. J Public Health Dent.

[8] Phantumvanit P, Songpaisan Y, Pilot T, Frencken JE (1996). Atraumatic restorative treatment (ART): a three-year community field trial in Thailand – survival of one-surface restorations in the permanent dentition. J Public Health Dent.

[9] van Dijken JW, Pallesen U (2015). Randomized 3-year clinical evaluation of Class I and II posterior resin restorations placed with a bulk-fill resin composite and a one-step self-etching adhesive. J Adhes Dent.

[10] de Amorim RG, Leal SC, Frencken JE (2012). Survival of atraumatic restorative treatment (ART) sealants and restorations: a meta-analysis. Clin Oral Investig.

[11] WHO – World Health Organization (2002). WHO Oral Health Data Bank.

[12] Gruebbel AO (1944). Post-War implications of fluorine and dental health: from the viewpoint of public health dentistry. Am J Public Health Nations Health.

[13] Löe H, von der Fehr FR, Schiött CR (1972). Inhibition of experimental caries by plaque prevention: the effect of clorhexidine mouthrinses. Scand J Dent Res.

[14] Kornman KS, Löe H (1993). The role of local factors in the etiology of periodontal diseases. Periodontol 2000.

[15] Frencken JE, Holmgren CJ (1999). How effective is ART in the management of dental caries?. Community Dent Oral Epidemiol.

[16] Roeleveld AC, Van Amerongen WE, Mandari GJ (2006). Influence of residual caries and cervical gaps on the survival rate of Class II glass ionomer restorations. Eur Arch Paediatr Dent.

[17] Bonifácio CC, Hesse D, Raggio DP, Bönecker M, van Loveren C, van Amerongen WE (2013). The effect of GIC-brand on the survival rate of proximal-ART restorations. Int J Paediatr Dent.

[18] Takanashi Y, Penrod JR, Lund JP, Feine JS (2004). A cost comparison of mandibular two-implant overdenture and conventional denture treatment. Int J Prosthodont.

[19] Kawai Y, Murakami H, Takanashi Y, Lund JP, Feine JS (2010). Efficient resource use in simplified complete denture fabrication. J Prosthodont.

[20] van Dijken JW, Pallesen U (2014). A randomized controlled three year evaluation of “bulk-filled” posterior resin restorations based on stress decreasing resin technology. Dent Mater.

[21] Elixhauser A, Halpern M, Schmier J, Luce BR (1998). Health care CBA and CEA from 1991 to 1996: an updated bibliography. Med Care.

[22] Hesse D, Bonifácio CC, Guglielmi CA, Bönecker M, van Amerongen WE, Raggio DP (2015). Bilayer technique and nano-filled coating increase success of approximal ART restorations: a randomized clinical trial. Int J Paediatr Dent.

[23] Innes NP, Evans DJ, Stirrups DR (2007). The Hall Technique; a randomized controlled clinical trial of a novel method of managing carious primary molars in general dental practice: acceptability of the technique and outcomes at 23 months. BMC Oral Health.

[24] Bonifácio CC, Hesse D, Bönecker M, Van Loveren C, Van Amerongen WE, Raggio DP (2013). A preliminary clinical trial using flowable glass-ionomer cement as a liner in proximal-ART restorations: the operator effect. Med Oral Patol Oral Cir Bucal.

[25] Boon CP, Visser NL, Kemoli AM, van Amerongen WE (2010). ART class II restoration loss in primary molars: re-restoration or not?. Eur Arch Paediatr Dent.

[26] Mickenautsch S, Yengopal V, Banerjee A (2010). Atraumatic restorative treatment versus amalgam restoration longevity: a systematic review. Clin Oral Invest.

[27] Raggio DP, Hesse D, Lenzi TL, Guglielmi CA, Braga MM (2013). Is Atraumatic restorative treatment an option for restoring occlusoproximal caries lesions in primary teeth? A systematic review and meta-analysis. Int J Paediatr Dent.

