# Supplementary material for: Effectiveness of conventional treatment using bulk-fill composite resin versus Atraumatic Restorative Treatments in primary and permanent dentition: a pragmatic randomized clinical trial
Source: BMC Oral Health. 2016 Aug 2;17:34. doi: 10.1186/s12903-016-0260-6 (PMC4970260; doi:10.1186/s12903-016-0260-6)
Supplement: Additional file 2: — Evaluation Criteria modified from Roeleveld et al. 2006. (DOCX 15 kb) [file 12903_2016_260_MOESM2_ESM.docx]

| Score Criteria | |
| --- | --- |
| 00 | Restoration present, correct |
| 10 | Restoration present, slight marginal defect/wear of surface (<0.5 mm). No repair needed |
| 11 | Restoration present, gross marginal defect/wear of surface (>0.5 mm). Repair needed |
| 12 | Restoration present, underfilled (>0.5 mm). Repair needed |
| 13 | Restoration present, overfilled (>0.5 mm). Repair needed |
| 20 | Secondary caries, discoloration in depth, surface hard and intact, caries within dentin. Repair needed |
| 21 | Secondary caries, surface defect, caries within dentin. Repair needed |
| 30 | Restoration not present. Repair needed |
| 31 | Restoration partly present (dentin exposed). Repair needed |
| 32 | Restoration partly present (dentine not exposed). No repair needed |
| 40 | Inflammation of the pulp; signs of dentogenic infection (abscesses, fistulae, pain complaints). Restoration might still be in situ. Extraction needed |
| 50 | Tooth not present because of extraction |
| 60 | Tooth not present because of shedding |
| 70 | Tooth not present because of extraction or shedding |
| 90 | Patient not present |
