# Supplementary material for: Effectiveness of conventional treatment using bulk-fill composite resin versus Atraumatic Restorative Treatments in primary and permanent dentition: a pragmatic randomized clinical trial
Source: BMC Oral Health. 2016 Aug 2;17:34. doi: 10.1186/s12903-016-0260-6 (PMC4970260; doi:10.1186/s12903-016-0260-6)
Supplement: Additional file 3: — Questionnaire adapted from Pani et al. (2014) [3]. (DOCX 18 kb) [file 12903_2016_260_MOESM3_ESM.docx]

1. Students responses on questions regarding their training and clinical ease in the use of GIC and composite resin.

| Theoretical knowledge of dental restorative materials: |  | My studies on GIC are more comprehensive |
| --- | --- | --- |
|  |  | My studies on composite are more comprehensive |
|  |  | My studies on both are equal |
| Pre-clinical training for handling dental restorative materials: |  | I have had more training with GIC |
|  |  | I have had more training with composite |
|  |  | My training for both is equal |
| How long does it take for you to prepare a simple class I restoration using GIC or composite? |  | Preparing for GIC is more time consuming than composite |
|  |  | Preparing for composite is more time consuming than amalgam |
|  |  | It takes me about the same amount of time for both |
| Which material do you personally prefer? |  | I prefer using GIC |
|  |  | I prefer using composite |
|  |  | It does not matter to me |

| In your opinion, what is the greatest drawback (negative aspect) of High Viscous Glass Ionomer Cement |  | Non-esthetic |
| --- | --- | --- |
|  |  | Adhesion to dental substract |
|  |  | Restoration Wear |
|  |  | Mechanical proprieties |
| In your opinion, what is the greatest drawback (negative aspect) of Composite |  | Micro-leakage |
|  |  | Technique sensitive |
|  |  | Takes more time |
|  |  | Poor clinical survival |

1. Differences between dentists in the influences affecting usage of GIC and composite resin.

| More lectures taken on this material |  | Resin Composite |
| --- | --- | --- |
|  |  | High Viscosity Glass Ionomer Cement |
| Instructors prefer that I use this material more often |  | Resin Composite |
|  |  | High Viscosity Glass Ionomer Cement |
| Patients prefer this material |  | Resin Composite |
|  |  | High Viscosity Glass Ionomer Cement |
| This material is easier for me to use and handle |  | Resin Composite |
|  |  | High Viscosity Glass Ionomer Cement |
| The properties of this material make it more ideal |  | Resin Composite |
|  |  | High Viscosity Glass Ionomer Cement |
| The cost of this material makes it a better choice |  | Resin Composite |
|  |  | High Viscosity Glass Ionomer Cement |
| Cavity preparation is much easier with this material |  | Resin Composite |
|  |  | High Viscosity Glass Ionomer Cement |

|  | YES | NO |
| --- | --- | --- |
| Do you believe GIC is a good substitute for composite? |  |  |
| In the mouth of a cooperative patient, do you believe composite restorations would last longer than amalgam ones? |  |  |
